# The Mouse Resistance Protein Irgm1 (LRG-47): A Regulator or an Effector of Pathogen Defense?

**DOI:** 10.1371/journal.ppat.1001008

**Published:** 2010-07-15

**Authors:** Julia P. Hunn, Jonathan C. Howard

**Affiliations:** Institute for Genetics, University of Cologne, Cologne, Germany; The Fox Chase Cancer Center, United States of America

The interferon-γ (IFNγ)–inducible IRG proteins are a distinctive cytoplasmic GTPase family encoded by about 20 genes in the C57BL/6 mouse [1]. All four IRG genes that have been knocked out (Irgm1, Irgm3, Irgd, Irga6) have caused more or less striking susceptibility phenotypes to *Toxoplasma gondii* ([2], [3] and O. Liesenfeld, I. Parvanova, J. Zerrahn, S-J. Han, F. Heinrich, et al., unpublished data). However, one single member of the IRG family, Irgm1 (formerly called LRG-47), stands out because it has additionally been implicated in a remarkable range of resistances in the mouse: resistance to *Trypanosoma cruzi* [4], *Leishmania major* [5], *Listeria monocytogenes* [2], *Mycobacterium tuberculosis* [6], *Mycobacterium avium* [7], *Chlamydia trachomatis* [8], and *Salmonella typhimurium* [9]. These are all intracellular but otherwise very different organisms—some are protozoa, some Gram-negative bacteria, some Gram-positive, some living inside a vacuole or phagosome, and some free in the cytosol. Thus, Irgm1 appears to have exceptional properties of disease resistance not shared by other members of the IRG family.

**Figure 1:**
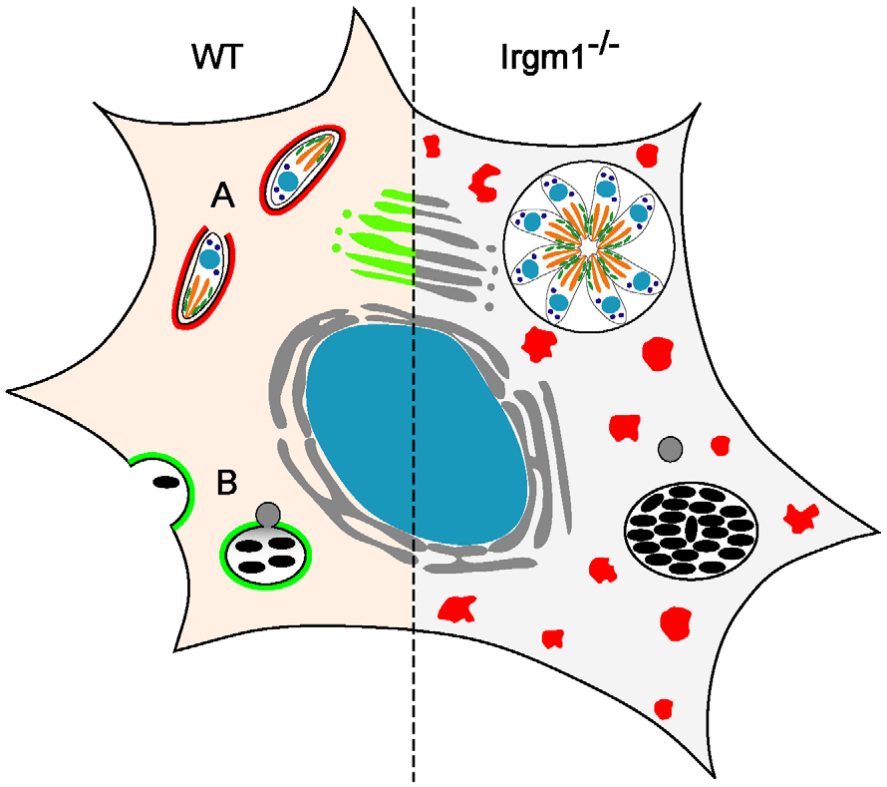
Irgm1 in cell-autonomous immunity. **(Left)** In the wild-type, IFNγ-treated cell, IRG proteins are induced and both *T. gondii* (A) and mycobacteria (B) are killed. Many IRG proteins accumulate around the *T. gondii* vacuole (indicated in red at (A)), while only the normally Golgi-associated Irgm1 (green) is thought to accumulate around the mycobacterial phagosome [6], [36]. There is little doubt that destruction of *T. gondii* is initiated by an IRG protein–mediated direct attack on the parasitophorous vacuole membrane [21]. It has been argued that Irgm1 on the mycobacterial phagosome membrane is directly responsible for fast acidification of the phagosome by lysosomal fusion ([6], indicated in grey at (B)) and perhaps also for initiation of autophagy [10]. **(Right)** Loss of Irgm1 results in loss of control of both *T. gondii* and mycobacteria. However, Irgm1 is one of three essential regulatory proteins belonging to the GMS subfamily of IRG proteins (Irgm1, Irgm2, Irgm3), that prevent premature activation of the GKS subfamily IRG proteins (Irga6, Irgb6, Irgd, etc.; red) in IFNγ-induced cells [19]. Loss of Irgm1 causes the normally markedly cytosolic GKS proteins (shaded red on the left) to form large, GTP-bound, non-functional aggregates (red dots) in IFNγ-induced cells [14] with striking cytopathic effects, especially on cells of the lymphomyeloid system [7], [15]. We argue that this, rather than loss of Irgm1 from the mycobacterial phagosome, is the main reason for the dramatic immune impairment of Irgm1-deficient mice, including loss of mycobacterial resistance.

Specific cell-autonomous resistance mechanisms have been attributed to Irgm1 in the context of mycobacterial resistance. Irgm1 has been considered to act by associating with the mycobacterial phagosomal membrane and accelerating lysosomal fusion [6] (Figure 1). There have also been suggestions that under certain conditions the protein can enhance the formation of autophagosomes that in turn control the pathogen [10]–[12]. These activities, related but distinct, could both be attractive candidates for a relatively direct mode of action of Irgm1 as a resistance protein.

These mechanisms for Irgm1 function are widely accepted, perhaps partly because of the importance of the diseases that they are supposed to control, but also because, right or wrong, they are immediately appreciable, plausible, proximal, cell-autonomous effects on the pathogen. However, optimism that there may be such direct explanations for the loss of mycobacterial resistance as a result of the loss of Irgm1 has apparently obscured an important literature on Irgm1 deficiency and activity that points in an entirely different direction.

Irgm1-deficient mice become strikingly leukopenic when infected with mycobacteria. Alan Sher and colleagues reported some years ago that the blood picture of young Irgm1-deficient adults is pretty normal, but collapses during infection [7]. They subsequently observed the same phenomenon for *Trypanosoma* infection [4]. A complete catalog has not yet been made, but we may infer that leukopenia is the rule when Irgm1-deficient mice are infected with any immunostimulatory pathogen. Indeed, induced lymphopenia seems also to arise following non-pathogenic immune stimuli since induction of experimental allergic encephalitis in Irgm1-deficient mice with myelin basic protein peptides, a well-established model for multiple sclerosis, resulted in similar leukocytic defects, in this case with a beneficial outcome for the disease [13]. Thus we ask, does susceptibility to mycobacteria (or *T. cruzi* or *Salmonella*) really have something to do with the proposed cell-autonomous mechanisms, autophagocytosis or reduced vacuole acidification, or is it due to the profound and generalized immunodeficiency that these organisms induce in Irgm1-deficient hosts?

Gregory Taylor and colleagues showed recently that mice that are not only Irgm1- but also Irgm3-deficient (that is, they have a doubly deficient IRG system) are no longer susceptible to *Salmonella* [14] (see Table 1). Furthermore, the authors cite a personal communication from John MacMicking that the mycobacterial susceptibility phenotype of Irgm1-single-deficient mice is also reversed in the same double knock-out. Thus the absence of Irgm1 cannot be the *direct* cause of the susceptibility in either of these cases. There must be a more complex explanation.

**Table 1:** Summary of cellular and systemic consequences of IRGM knock-outs.

Genotype	
**Wt**	Normal expression and regulation of induced effector IRG proteinsNo cytopathic consequences for cellular functionHeightened cell-autonomous immunity via IRG proteinsResistance against a wide range of intracellular pathogens^a^
**Irgm1^−/−^**	Incomplete regulation of induced effector IRG proteinsCytosolic aggregates of IRG proteins with cytopathic consequences:- Stem cell exhaustion- Massive leukopenia- Systemic immune deficiency- Macrophage dysfunction: reduced motility, impaired adhesiveness, reduced phagosome acidification, multiple cell-autonomous immune deficienciesSusceptibility to multiple pathogens including mycobacteria, *Salmonella*, *Trypanosoma*, and *Leishmania* in addition to *C. trachomatis* and *T. gondii*
**Irgm1/m3^−/−^**	Incomplete regulation of induced effector IRG proteinsStrongly reduced expression of effector IRG proteinsCytosolic aggregates of IRG proteins with enhanced clearance and no cytopathic consequences:- No stem cell exhaustion- No leukopenia- No systemic immune deficiency- No cell-autonomous dysfunction except loss of IRG-dependent immunitySusceptibility only to *T. gondii* and *C. trachomatis* a

This table summarizes the arguments presented, documented, and referenced in the accompanying article. Each panel can be read from top to bottom as a causal chain. Thus, Irgm1 deficiency results in incomplete regulation of induced effector GKS IRG proteins, which results in build up of cytosolic aggregates, and these in turn have cytopathic consequences. For Irgm1 deficiency, the causal chain is long and ends up with major systemic and cell-autonomous immunodeficiency. In wild-type cells, the causal chain is adaptive and leads to increased cell-autonomous immune competence, while in the Irgm1/Irgm3 double-deficient cells the causal chain heading towards cytopathy is truncated by the rapid clearance of the IRG protein aggregates. The consequences of Irgm1 deficiency are cellular as well as systemic and result in whole-animal immune failure.

a The range of pathogens genuinely controlled by the IRG system of mice is unclear. At present, *T. gondii* and *C. trachomatis* stand out, but it is not known what these two pathogens have in common that renders them susceptible to IRG-mediated immunity, nor what the other organisms lack or possess that renders them resistant.

Why do Irgm1-deficient animals rapidly develop a lymphomyeloid deficiency after infection or autoimmune stimulation? There seems to be reduced proliferative potential in the lymphomyeloid system that becomes acute after immune activation. It was shown recently that this affects the hematopoietic stem cell (HSC) as well as more peripheral lymphoid compartments [15]. The functional impairment depends absolutely on the presence of IFNγ and the integrity of its signal transduction pathway. If these are impaired or impeded, the Irgm1-dependent hematopoietic and lymphopoietic failures are reversed, as is susceptibility to infection by mycobacteria ([16], [17]; Margaret Goodell, personal communication). Thus, absence of Irgm1 is not *in itself* responsible for the hemopoietic and immune failures. Rather, it is the rest of the IFN response that is causing the problem in the absence of Irgm1. Stressing this point, Irgm1-deficient mice infected with a pathogen that stimulates only a Th2 response (*Schistosoma mansoni*), and therefore essentially no IFNγ production, show normal resistance and no lymphoid abnormalities [16]. Which of the thousand or so IFNγ-regulated transcripts is responsible for this mysterious effect? The double knock-out of Irgm3 and Irgm1 seems to tell us the interesting answer, that the problem with Irgm1 deficiency is connected with the presence of the rest of the IRG family of proteins. What can that problem be?

We showed that the IRG proteins fall into two groups, the GKS and the GMS sub-families, based on the sequence of their nucleotide binding domains [18]. More recently, we showed that the three GMS proteins, Irgm1 (LRG-47), Irgm2 (GTPI), and Irgm3 (IGTP), are essential regulators of the GTPase cycle of the GKS proteins, binding to these in the GDP-bound state and acting as attenuators, preventing premature activation of GKS proteins by the binding of GTP [19]. If even only one of the three GMS regulator proteins is absent, the GKS effector proteins form GTP-bound aggregates in the cell [19], [20]. Under these conditions, the GKS proteins can no longer exercise their only confirmed function of relocating to the *T. gondii* vacuole and initiating vacuolar disruption [21] (see Figure 1). For still unclear reasons, all three GMS proteins must be present for normal behavior of the GKS proteins.

It was shown some time ago that unregulated GKS proteins can interfere with cell proliferation. Douglas Carlow and colleagues attempted to generate fibroblast cell lines constitutively expressing the GKS effector IRG protein Irgb6 in the absence of IFNγ, and therefore in the absence of the three GMS proteins [22]. These cell lines regularly lost expression of the protein, and they showed only limited stability even when expressing very low levels of the protein. Constitutive expression of Irga6 in cells in the absence of IFNγ led to the formation of protein aggregates associated with marked pathological expansion of the endoplasmic reticulum lumen, though apparently without interfering with cell proliferation of mouse 3T3 fibroblasts [19]. It is worth mentioning that expression of individual GMS proteins has no detectable cytopathic or cytostatic effect on cells growing in culture ([19] and J. Hunn, S. Könen-Waisman, J. Howard, unpublished data).

We can therefore propose the following preliminary scenario for the Irgm1-deficient mouse. In the absence of induced IFNγ production, the mouse appears relatively normal. However, for unclear reasons, there is constitutive expression of many IRG genes in HSCs [23]–[25]. In the absence of Irgm1, this expression would be expected to result in unregulated cytoplasmic aggregates of GKS proteins. These are presumably cytostatic or cytopathic in the HSC population, resulting in continuous turnover and concomitant near exhaustion of the stem cell pool, leaving little residual potential to respond to hematopoietic stress [15]. In the periphery, infection rapidly induces IFNγ, which in turn induces the IRG protein response in lymphoid and other cells. As in HSC, Irgm1 deficiency results in the formation of intracellular aggregates of unregulated GKS proteins [14], [19], [20]. These aggregates are presumably cytostatic or cytopathic for cells of the lymphomyeloid system, perhaps especially for replicating cells through inhibition of the ubiquitin-proteasome system [26], resulting in the observed infection-induced leukopenia and a generalized immunodeficiency. It seems that IRG aggregate formation must also be toxic for interphase lymphocytes to explain the generalized lymphopenia. We would argue that the deposition of aggregates in IFNγ-induced cells is responsible for the autophagic anomalies observed in Irgm1-deficient T lymphocytes [16].

Consistent with this scenario, Taylor and colleagues showed aggregates of GKS proteins (Irgb6 and Irga6) in bone marrow–derived macrophages from Irgm1 knock-out mice after in vitro stimulation with IFNγ [14]. However, they also observed aggregates in IFNγ-induced cells from Irgm3 knock-outs and Irgm1/Irgm3 double knock-outs, neither of which show a significant lymphopenia nor susceptibility phenotype to *Salmonella* or mycobacteria infection [6], [9]. At first glance, this latter observation seems to argue against the idea that protein aggregates are responsible for the cytopathic sequelae of Irgm1 loss [14]. However, aggregates forming as a result of Irgm1 deficiency may well be qualitatively distinct from, and more cytotoxic than, those resulting from Irgm3 deficiency. We have shown that all three GMS regulators are required for complete GKS control and have hypothesized that each is required for GKS regulation on a different group of endomembranes [19]. Thus, Irgm1 deficiency may lead preferentially to GKS aggregation on Golgi and endolysosomal membranes, where Irgm1 is normally localized [27], [28], while aggregates due to Irgm3 deficiency form preferentially on endoplasmic reticulum membranes, where Irgm3 is normally localized [29]. There is already evidence that Irga6 and Irgb6 may be preferentially regulated to a different extent by individual GMS proteins [19]. Such distinctions may well result in different cytopathic phenotypes for different GMS deficiencies depending on the level and subcellular localization of dysregulation. Taylor and colleagues also noticed that there was a reduced amount of GKS IRG proteins in IFNγ-induced Irgm3-deficient macrophages compared with the wild-type or Irgm1-deficient cells [14]. In the Irgm1/Irgm3 double knock-out cells the amount of GKS protein was very greatly reduced. This is presumably due to a substantially reduced half-life of aggregated GKS protein. Thus, there may be a quantitative as well as a qualitative reason for the heightened cytopathic effects of Irgm1 deficiency compared with Irgm3 deficiency. An alternative view, that Irgm3 is cytopathic in the absence of Irgm1, we consider less convincing. There is no a priori basis for the supposition, and cells expressing Irgm3 alone, by transfection, show no cytopathic or proliferative anomalies, and the protein does not form intracellular aggregates ([19], [29] and J. Hunn, S. Könen-Waisman, J. Howard, unpublished data).

A plausible interpretation of the Irgm1 phenomenon now runs like this. Infection, for example by mycobacteria, induces a high level of IFNγ, which in turn induces high levels of IRG proteins. In the absence of Irgm1, the GTPase cycle of the remaining IRG proteins cannot be properly controlled. This results in the formation of large IRG protein aggregates that in certain cell types of the hematopoietic and lymphoid systems are cytopathic or cytostatic, causing a generalized lymphopenia. Losing Irgm3 and Irgm1 together causes rapid clearance of the aggregates and relieves the cytopathic phenotype. Thus, in the Irgm1/Irgm3 double knock-out the immune picture essentially returns to normal. In a recent note, Feng and colleagues have also proposed that at least part of the Irgm1 deficiency phenotype is due to the loss of a regulatory function [17]. However, our interpretation differs significantly from theirs. They propose a specific role for Irgm1 in the maintenance of T cell survival following IFNγ induction, while we view the function of Irgm1 to be confined to its regulatory activity in the GTPase cycle of the GKS IRG proteins and the prevention of aggregation. Their position, attributing a positive regulatory effect by Irgm1 inhibiting an autophagy-mediated cell death, seems to offer no explanation for the loss of the Irgm1 deficiency phenotype in the Irgm1/m3 double-deficient mouse.

Resistance to *Toxoplasma* is completely lost in the Irgm1 knock-out [2], and this could of course as easily be due to the generalized immunodeficiency as to the loss of a key IRG protein function. However, resistance to *Toxoplasma* does not return in the Irgm1/Irgm3 double knock-out [14]. Furthermore, loss of Irgd or Irga6, both GKS proteins, also leads to a *Toxoplasma* susceptibility phenotype without any lymphopenia or generalized immunodeficiency ([2], [21] and O. Liesenfeld, I. Parvanova, J. Zerrahn, S-J. Han, F. Heinrich, et al., unpublished data). The conclusion is that the IRG proteins really do mediate resistance against *Toxoplasma* in mice. It is a good bet that the ability of multiple IRG proteins to relocalize to the *T. gondii* parasitophorous vacuole membrane, causing its disruption and killing the parasite [21], [30], indicates the essential mechanism by which IRG proteins operate against this pathogen.

We conclude that the adaptive role of Irgm1 in mice is connected to its activity in the regulation of the GKS members of the IRG protein family. *T. gondii* is probably an important pathogen for mice because of the recent predominance of the domestic cat as definitive host, and it may therefore be that resistance to this parasite is driving the function of the IRG system in the mouse. Recent results from Jörn Coers and colleagues show that the IRG system may also be directly active against *C. trachomatis* [8], [31]. However, we consider it highly unlikely that Irgm1 has any adaptive function at all in resistance against most of the other pathogens attributed to it. Certainly mycobacteria and *Salmonella* can now be explicitly excluded [6], [9], and there is every reason to suppose that most if not all the others except *T. gondii* and *C. trachomatis* will go the same way.

It is important to look back on the experiments that attributed specific cell-autonomous activities to Irgm1 to account for its role in resistance to mycobacteria. If resistance to mycobacteria or *Salmonella* really has nothing to do with IRG proteins, why does Irgm1 relocalize to the mycobacterial phagosome, and why would acidification of the phagosome be reduced in Irgm1-deficient cells [6] (see Figure 1)? Most of the relevant experiments were conducted on macrophages derived from the Irgm1-deficient strain, so it is the properties of macrophages that should be considered. As to the first point, it was shown some years ago that Irgm1 relocalizes to latex bead phagosomes in macrophages [27], so this step has nothing necessarily to do with mycobacterial infection. To the second point, Taylor and colleagues have described striking cell-autonomous abnormalities in the motility and adhesiveness of macrophages derived from Irgm1-deficient mice [9], [14], [32]. These defects are completely reversed in the Irgm1/Irgm3 double knock-out [14]. In view of the hematopoietic abnormalities in the Irgm1-deficient mice, macrophage development and differentiation are probably also disturbed. Aggregate formation in Irgm1-deficient macrophages [14] may also have direct cytopathic consequences for many aspects of macrophage activity, including lysosomal function, perhaps as a result of autophagy, constitutively stimulated by the presence of IRG protein aggregates [33]. Therefore, a direct comparison between the cell-autonomous properties, such as phagocytic vacuole acidification and induction of autophagy, of Irgm1-deficient and wild-type macrophages is probably not valid. A direct analysis of phagosome and autophagosome function in the single and double GMS knock-outs would clarify whether some direct cell-autonomous function can be attributed to Irgm1.

It is also interesting to revisit the specificity control introduced by Taylor and colleagues to indicate that the immune deficiency due to Irgm1 was not universal, namely that resistance to mouse cytomegalovirus (MCMV) was normal [2], [3]. Resistance to MCMV does not depend on T cells but is largely mediated by natural killer cells, which require cytokine-mediated activation to develop full functional activity [34], [35]. This cell type may be less vulnerable to the cytopathic consequences of Irgm1 deficiency than T cells and HSCs.

It is important to emphasize that while the present view can account for much of the complexity of the observations on Irgm1 deficiency, it remains possible that Irgm1 may have additional “autonomous” activities of its own, perhaps in the control of autophagy. It now seems unlikely that this will be true for immunity against mycobacteria or *Salmonella* since this appears to be normal in the absence of Irgm1 so long as Irgm3 is missing too, but these, of course, do not exhaust the universe of intracellular pathogens. There is much experimental work left to do to assess the validity and completeness of this revision of view about how the IRG proteins fulfill their function. It is a complex argument, but it hangs together reasonably well and offers a broad and satisfying explanation for most, if not all, of the properties of the Irgm1-deficient mouse. Above all, however, the IRG system must be understood as a highly regulated, highly coordinated system of proteins where the properties of single-gene knock-outs may be misleading.
